# Supplementary figures and images for: Different artificial feeding strategies shape the diverse gut microbial communities and functions with the potential risk of pathogen transmission to captive Asian small-clawed otters (Aonyx cinereus)
Source: mSystems. 2024 Nov 27;9(12):e00954-24. doi: 10.1128/msystems.00954-24 (PMC11651104; doi:10.1128/msystems.00954-24)

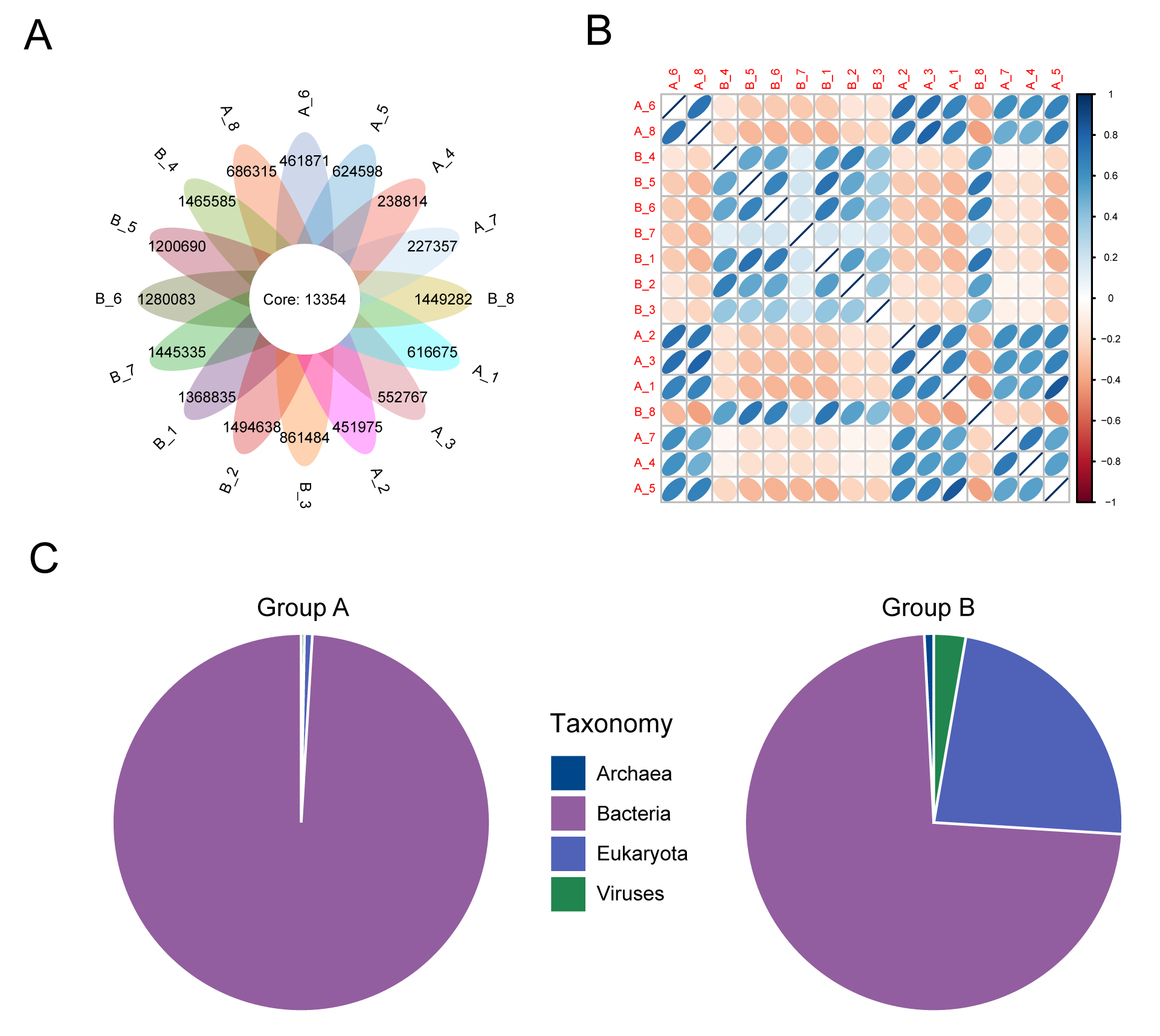

Supplement: Fig. S1 — The distributions of non-redundant unique genes in samples of two groups. [file msystems.00954-24-s0001.tif]

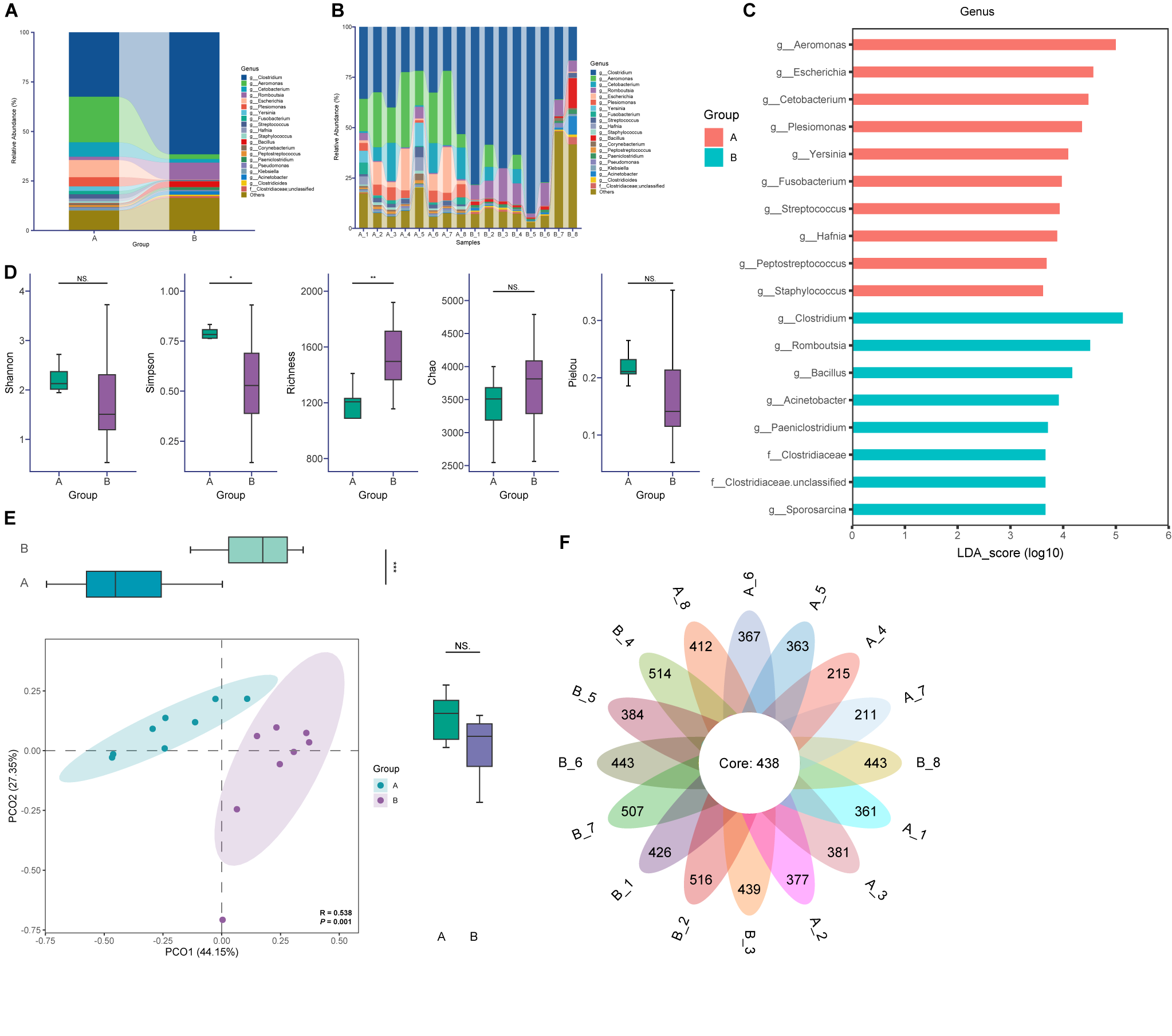

Supplement: Fig. S2 — The compositions and differences of fecal microbial communities of otters from two groups in genus level. [file msystems.00954-24-s0002.tif]
