# Supplementary material for: Different artificial feeding strategies shape the diverse gut microbial communities and functions with the potential risk of pathogen transmission to captive Asian small-clawed otters (Aonyx cinereus)
Source: mSystems. 2024 Nov 27;9(12):e00954-24. doi: 10.1128/msystems.00954-24 (PMC11651104; doi:10.1128/msystems.00954-24)
Supplement: Legends — Supplemental figure and table legends. [file msystems.00954-24-s0003.docx]

**Supplemental Figures for manuscript of “Different artificial feeding strategies shape the diverse gut microbial communities and functions with the potential risk of pathogen transmission to captive** **Asian small-clawed otters (*Aonyx cinereus*)”**

Yuanda Gao ^a^, Hangyu Zhang ^a^, Dapeng Zhu ^b*^, Long Guo ^a*^

^a^ State Key Laboratory of Herbage Improvement and Grassland Agro-Ecosystems, College of Pastoral Agriculture Science and Technology, Lanzhou University, Lanzhou, 730020, China.

^b^ Foping National Nature Reserve, Hanzhong 723000, China

* Corresponding author.

E-mail address: [guolong@lzu.edu.cn](mailto:guolong@lzu.edu.cn) (L. Guo)

**Figure legends**

**FIG S1.** The distributions of non-redundant unique genes in samples of two groups.

(A) Flower plot of Venn analysis of the distributions of unique genes in 16 samples.

(B) The correlation analysis between samples based on the distribution of unique genes.

(C) Classification of unique genes into at superkingdom level.

**FIG S2.** The compositions and differences of fecal microbial communities of otters from two groups in genus level.

(A) The top 20 gut bacteria average relative abundances at genus level in group A and group B.

(B) The top 20 gut bacteria relative abundances at genus level in each sample.

(C) The LDA score (log 10) of differential bacteria at genus level (LDA score > 3.5 and KW test *P*-value < 0.001).

(D) The α diversity analysis at genus level including Shannon index, Simpson index, Richness index, Chao1 index, Pielou index.

(E) Principal-coordinate analysis (PCoA) plot of bacteria community at genus level based on Bray‒Curtis distance (n=8), **P*<0.05, ***P*<0.01 by independent-samples T test.

(F) Flower plot of Venn analysis of bacteria genera number in 16 samples.

**Table legends**

**Table S1.** The taxonomy and abundance information of taxa belonging to Eukaryota superkingdom.

**Table S2.** Metagenome-assembled genomes (MAGs) taxonomy information.

**Table S3.** Antibiotic resistance genes (ARGs) information.
